# Tryptophan-centered metabolic alterations coincides with lipid-mediated fungal response to cold stress

**DOI:** 10.1016/j.heliyon.2023.e13066

**Published:** 2023-01-21

**Authors:** Yonghong Chen, Xiaoyu Yang, Longlong Zhang, Qunfu Wu, Shuhong Li, Jianghui Gou, Jiangbo He, Keqin Zhang, Shenghong Li, Xuemei Niu

**Affiliations:** a State Key Laboratory for Conservation and Utilization of Bio-Resources in Yunnan, Key Laboratory for Southwest Microbial Diversity of the Ministry of Education, Yunnan University, Kunming 650032, China; b State Key Laboratory of Phytochemistry and Plant Resources in West China, Kunming Institute of Botany, Chinese Academy of Sciences, Kunming 650204, China; c Kunming Key Laboratory of Respiratory Disease, Kunming University, Kunming 650214, China

**Keywords:** *Thermomyces dupontii*, Prenylated indole alkaloids, Tryptophan, Lipids, Cold stress, Tryptophan metabolism, PIAs, prenyl indole alkaloids, TEM, transmission electron microscope

## Abstract

Tryptophan and its derived metabolites have been assumed to play important roles in the development and survival of organisms. However, the links of tryptophan and its derived metabolites to temperature change remained largely cryptic. Here we presented that a class of prenyl indole alkaloids biosynthesized from tryptophan dramatically accumulated in thermophilic fungus *Thermomyces dupontii* under cold stress, in which lipid droplets were also highly accumulated and whose conidiophores were highly build-up. Concurrently, disruption of the key *NRPS* gene involved in the biosynthesis of prenyl indole alkaloids, resulted in decreased lipid and shrunken mitochondria but enlarged vacuoles. Moreover, the Fe^3+^ and superoxide levels in *ΔNRPS* were significantly increased but the reactive oxygen species lipid peroxidation and autophagy levels decreased. Metabolomics study revealed that most enriched metabolites in *ΔNRPS* were mainly composed of tryptophan degraded metabolites including well known ROS scavenger kynurenamines, and lipid-inhibitors, anthranilic acid and indoleacetic acid, and free radical reaction suppressor free fatty acids. Transcriptomic analysis suggested that the key gene involved in tryptophan metabolism, coinciding with the lipid metabolic processes and ion transports were most up-regulated in *ΔNRPS* under stress. Our results confirmed a lipid-mediated fungal response to cold stress and unveiled a link of tryptophan-based metabolic reprogramming to the fungal cold adaption.

## Highlights

• Thermophilic fungus *T. dupontii* under cold stress accumulated lipid and tryptophan-derived metabolites, prenyl indole alkaloids (PIAs).
• PIAs deletion under cold stress caused increased Fe^3+^ level but decreased lipid level.
• Lipid inhibitor indole acid and ROS scavenger kynurenamines were accumulated in the mutant without PIAs.
• Concurrently, free fatty acids including unsaturated ones, were accumulated in the mutant without PIAs under cold stress.
• The most up-regulated gene in the mutant without PIAs under cold stress was responsible for the first step from tryptophan to kynurenamines.

## Graphical abstract

The mechanism by which organisms respond to temperature changes via biosynthesis of small-molecules remained largely cryptic. We reported that a class of tryptophan-derived natural products via non-ribosomal peptide synthase (NRPS) pathway, prenyl indole alkaloids (PIAs), accompanied the lipid formation in thermophilic fungus *T. dupontii* under cold stress. PIAs deletion increased Fe^3+^ level and initiated tryptophan metabolism to yield lipid inhibitors and reactive oxygen species (ROS) suppressors. Concurrently, a large amount of unsaturated and saturated free fatty acids with free radical scavenging ability were also accumulated. Our findings confirmed that lipid mass under cold stress should be an inevitably boosting risk for the fungus and unveiled two alternative metabolite-lipid-cold relationships, which provided a new insight into the ecological and biological functions of tryptophan-associated metabolites.

**Figure:**
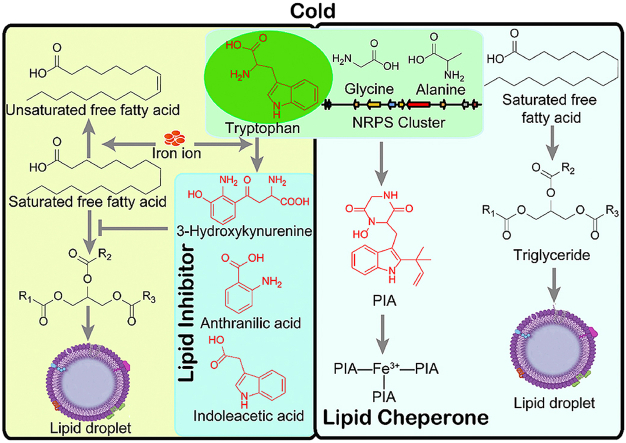

## 1 Introduction

Temperature change displays a paramount impact on all the forms of lives and change in lipid level for adaption to temperature change is a general phenomenon in organisms [1]. Moreover, metabolic changes have been found to be associated with and useful for the development and survival of the producing organisms in adaption to environment changes [2]. However, the link of metabolite change to temperature change and lipid level still remain largely unclear.

Tryptophan is a naturally-occurring essential amino acid and plays a rate-limiting role during protein synthesis because, among all amino acids, it has the lowest overall concentration in the human body [3]. Tryptophan metabolism leads to production of the well-known neuromodulator kynurenic acid and predominant natural auxin indoleacetic acid [4,5]. Moreover, tryptophan is a common building block for biosynthesis of complex metabolites with diverse structures and biological activities. Prenylated indole alkaloids (PIAs) are a family of natural products biosynthesized from tryptophan and a second amino acid constitute a well-known class of fungal metabolites with diverse chemical structures and a wide range of biological and pharmacological activities [6,7]. They are widely distributed in filamentous fungi, especially in the genera *Penicillium* and *Aspergillus* of Ascomycota [6,7]. However, few studies on the link of tryptophan to temperature change and of the well-known tryptophan derived metabolites to a cold have been reported.

Our another study displayed that predominant thermophilic fungus *Thermomyces lanuginosus* applied a major polyketide metabolite dihydroxynaphthalenes (DHN) to act as cell wall reinforcer against mass lipid accumulation and as reactive oxygen species (ROS) scavenger against massive lipid peroxidation to survive ferroptosis induced by a cold [8]. We also found a melanin-mediated low lipid mechanism against a cold-induced ferroptosis in mice. *Thermomyces* genus has only two species, *T.* (TL) and *T. dupontii* (TD) [9]. TL and TD overall share similar biosynthetic gene clusters with similar typical characteristics [10,11]. Interestingly, *T. lanuginosus* has commonly been reported to be the most dominant thermophilic fungus in diverse geothermal systems while its sister species *T. dupontii* was not [9,12]. What makes this obvious difference between these two-sister species in *Thermomyces* remained largely unknown.

Comparison of chemical profiles between the two *Thermomyces* species revealed that these two strains displayed completely different metabolic profiles. No DHN like metabolites were detected in *T. dupontii*, though these two fungi shared the unique bacterial-like PKS-NRPS hybrid thermolides [13]. Our pervious study suggested a series of prenyl indole alkaloids (PIA) in *T. dupontii* [13,14]. The ability of an individual fungus to respond to permissive environmental cues also depends on its metabolic stage [15,16]. This interaction between environmental and metabolic processes creating diversity in chemical responses.

In this work, we compared the phenotypic, metabolic and transcriptional responses of thermophilic fungus *T. dupontii* to two temperatures, a minimum growth temperature at 37 °C and an optimal growth temperature at 50 °C. Chemical investigations led to isolation and characterization of a series of PIA, which was involved in melanin biosynthesis induced by a cold. A combination of homologous recombination genetic manipulation and chemical screening led to successful construction of mutant Δ*NRPS* devoid of PIA. Collective bioassays revealed that Δ*NRPS* at low temperature displayed increased survival rate and vitality. Electron microscopy analysis displayed dramatically decreased lipid droplets and lack of mitochondrial cristae and membranes in Δ*NRPS*, a diagnostic characterization for ferroptosis. A series of ferroptosis assays revealed that lack of PIA caused a cascade of detrimental changes, leading to ferroptosis and PIA precursors and their derivatives acted a defense system against lipid mediated ferroptosis at low temperature.

## 2 Results

### 2.1 Accumulation of a series of PIAs biosynthesized from tryptophan in *T. dupontii* under cold stress

**Fig. 1:**
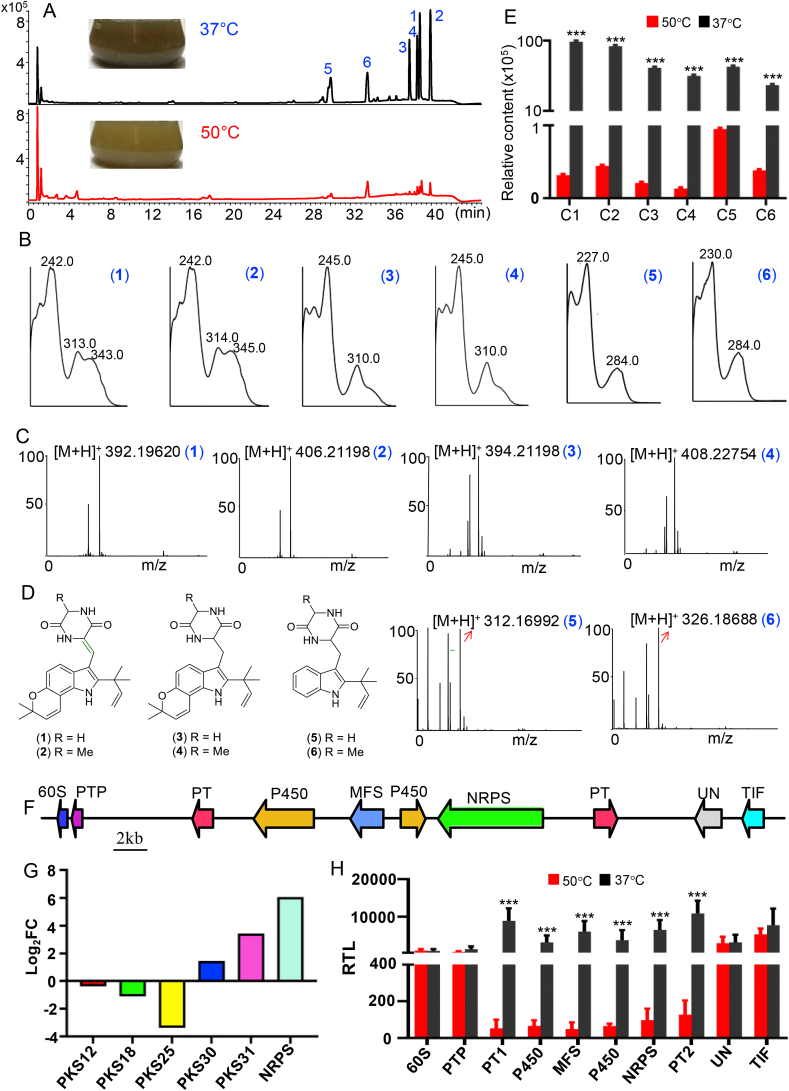
Accumulation of pigment PIAs, talathemophilins A–F (1–6) biosynthesized from tryptophan in *T. dupontii* at minimum growth temperature 37 °C, and characterization of the gene cluster and key genes involved in PIA biosynthesis. (A) Metabolic analysis of extracts of *T. dupontii* at optimal growth temperature 50 °C and minimum growth temperature 37 °C with six main peaks (1–6) at 37 °C with HPLC profiles. (B) Quantitative analysis of peaks (1–6) in the HPLC profiles of *T. dupontii* between at optimal growth temperature 50 °C and minimum growth temperature 37 °C. (C) The UV spectra for the six main peaks (1–6). (D) The HRMS for the six main peaks (1–6). (E) Structures of talathemophilin A–F (1–6). (F) Organization of the talathemophilin biosynthesis gene cluster in *T. dupontii.* 60S: 60S ribosomal protein; Phosphatase: protein-tyrosine-phosphatase; PT: aromatic prenyltransferase; MFS: MFS toxin efflux pump; TIF: Translation initiation IF2. (G) The relative transcriptional levels (RTL) of biosynthetic genes in *T. dupontii* at 37 °C vs. at 50 °C. RTL in log_2_FC (Fold change) value. (H) The relative transcriptional levels of genes in *TPS-NRPS* gene cluster in *T. dupontii* at 37 °C vs. at 50 °C. RTL in log_2_FC (Fold change) value.

*T. dupontii* demonstrated significantly darker yellow-green at 37 °C than at 50 °C (Fig. 1A). Metabolic analysis of the ethyl acetate extracts showed that six main peaks in the fungus at 37 °C exhibited (Fig. 1A) 93.6, 62.7, 89.3, 115.7, 20.2, and 30.4 fold higher than those in the fungus at 45 °C (Fig. 1B), respectively. The metabolites 1–5 displayed the ultraviolet (UV) absorptions quite similar to those of known talathermophilins A-E (1–5) reported from the same species (Fig. 1C) [17]. Combined with HPLC-HRMS (Fig. 1D), we identified five of the main products of *T. dupontii* as five PIAs (1–5) [17]. The sixth main product shared the same UV absorptions at 230 and 284 nm, similar to those of talathemophilin E (5) (Fig. 1C). Combined with a quasi-molecular ion peak at *m/z* 326.18688 [M+H]^+^ for a molecular formula of C_19_H_23_N_3_O_2_ in the positive HRESI spectrum (Fig. 1D), methyl talathemophilin E was assigned as 6 and named talathemophilin F (Fig. 1E). PIAs were biosynthesized from tryptophan with another amino acid to form the 2,5-diketopiperazine followed by prenylation. Interestingly, only a very small group of amino acids (glycine, alanine, proline and its derivatives) could be naturally chosen as a starting building block to condensation with tryptophan. Metabolites 1–6 were derived from tryptophan with alanine or glycine. Among them, metabolites 1 and 2 were just dehydro analogues of 3 and 4, respectively (Fig. 1E). Metabolites 5 and 6 were the precursors for metabolites 1–4, which had one more prenyl unit at the phenyl ring of indole unit.

### 2.2 Characterization of gene *NRPS* and construction of the mutant devoid of PIAs

Phylogenetic analysis using the maximum likelihood algorithm showed that the NRPS clustered most closely with NRPS proteins from *Aspergillus* and *Penicillium* (Fig. S1). Transcriptional analysis of the fungus at 37 °C vs. at 50 °C revealed that one gene assigned to *NRPS* (Fig. 1F), an analogue of the *NRPS* gene putatively responsible for PIAs [18], was the most significantly up-regulated in all the biosynthetic core genes at 37 °C vs. at 50 °C (Fig. 1G, Tables S1 and S2). We found that five genes in the same *NRPS* cluster, encoding two prenyltransferases, two P450, and one MFS toxin efflux pump, also responsible for PIA biosynthesis, were also highly up-regulated with about 57–167 fold higher at 37 °C than at 50 °C (Fig. 1H, Tables S1 and S2), suggesting that the *NRPS* cluster in *T. dupontii* was the paramount biosynthetic gene cluster in fungal response to chronic low temperature at 37 °C. A *NPKS* knockout strain (Δ*NRPS*) was finally achieved through a modified homologous recombination system for *T. dupontii* (Fig. S2) [10]. Phenotypic and metabolic analysis revealed that Δ*NRPS* was incapable of producing PIAs and their derived pigments (Fig. S3).

### 2.3 Functions of PIAs in fungal conidiation and spore germination rate under cold stress

**Fig. 2:**
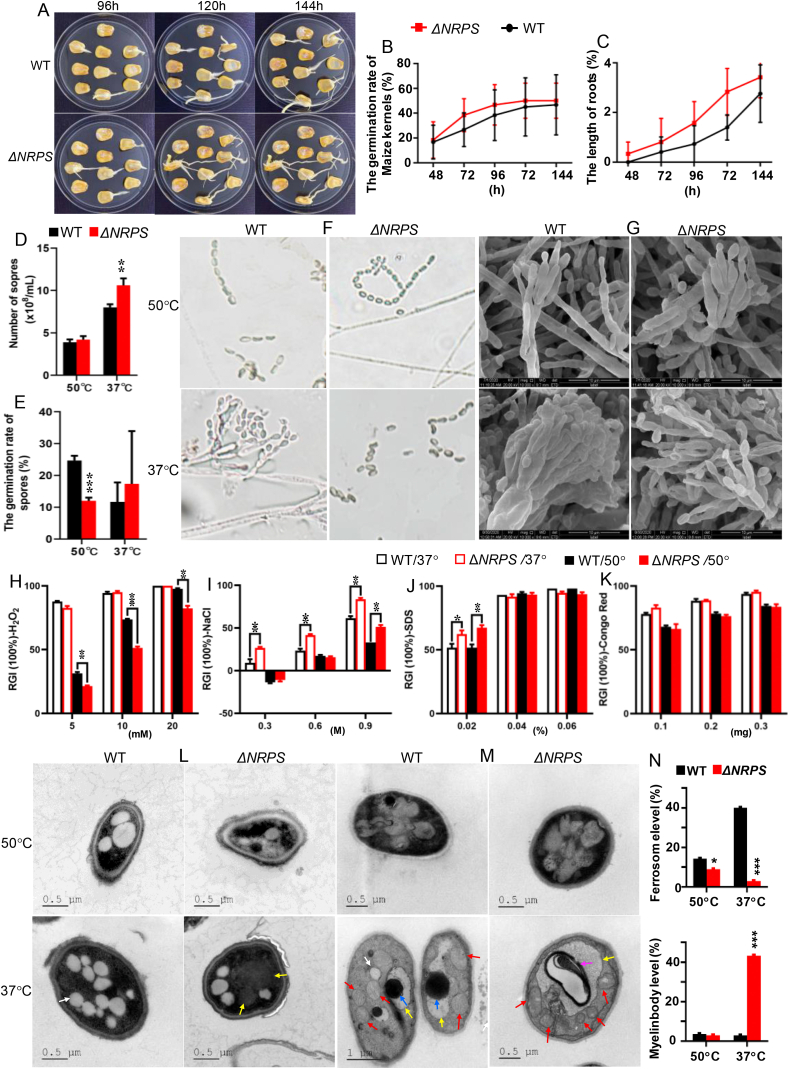
Effect of PIAs on fungal vitality, growth, conidia formation, germination and physiology. (A) Comparison of fungal effects on maize kernel germination rates and root growth between Δ*NRPS* and WT at 37 °C. (B–C) Quantitative analysis of the maize kernel germination rates and root growth between between Δ*NRPS* and WT groups. (D–E) Comparison of conidia and spore germination rate between Δ*NRPS* and WT at 50 °C and 37 °C. Error bars are the standard deviation. (F) Comparison of conidiophores and conidia between Δ*NRPS* and WT under a microscope. Bar = 20 μm. (G) Comparison of conidiophores and conidia between Δ*NRPS* and WT under scanning electron microscopy. Bar = 0.5–1 μm. (H–K) Comparison of fungal multi-responses between Δ*NRPS* and WT at 50 °C and 37 °C to oxidant 5–20 mM H_2_O_2_ (H), osmotic agent 0.3–0.9 M NaCl (I), and two cell-wall-perturbing agents 0.2–0.6% SDS (J) and 0.1–0.3 mg/mL Congo red (K). (L–M) Comparison of the infrastructures inside conidia (L) and mycelia cells (M) between Δ*NRPS* and WT under transmission electronic microscopy (TEM). White arrow refers to lipid droplet; red arrow refers to mitochondrion; yellow arrow refers to vacuole; blue arrow refers to black tight body (most likely ferrosome), rose arrow refers to myelin body. (N) Quantitative analysis of the ferrosomes and myelin bodies between Δ*NRPS* and WT at 37 °C. (For interpretation of the references to color in this figure legend, the reader is referred to the Web version of this article.)

Previous study suggested that thermophilic fungi dominated the mycoflora in the piled up corn grains [9,12]. Maize kernel bioassay revealed that Δ*NRPS* at 37 °C exhibited obvious increase in spore germination rate and colonization on maize, and in promoting maize seed germination and sprout growth (Fig. 2A). The maize germination rate in Δ*NRPS* group increased by 44% at 72 h and the seedling growth treated with Δ*NRPS* increased by 32% at 72 h (Fig. 2B and C). Δ*NRPS* at 37 °C displayed significance increases in conidial formation and spore germination rate with increase rates of 32% and 98%, respectively, compared to WT at 37 °C (Fig. 2D and E). Further analysis with microscopic and scanning electron microscopy (SEM) revealed that WT at 37 °C displayed some large and stout penicillin structures (5%), which were not observed in WT at 50 °C and Δ*NRPS* at both temperatures, suggesting that PIAs might be involved in binding the separate conidiophores into penicillin structures at low temperature (Fig. 2F and G). It seemed that the lack of PIAs as conidiophore binders might contribute to increased conidial spores in Δ*NRPS*. We assumed that similar to its sister strain *T. lanuginosus* that used a major metabolite as cell wall reinforcer against a cold in order to low the need for lipid mass [8], *T. dupontii* preferred to use PIA as conidiophores reinforcers to shield against low temperature-mediated lipid mass.

### 2.4 Increased responses of Δ*NRPS* to oxidant and ion stresses under cold stress

Δ*NRPS* exhibited significantly increased sensitivity to 5–20 mM H_2_O_2_ and increased resistance to 0.9 M NaCl compared to WT at 37 °C (Fig. 2H and I), while no significant differences in response to SDS and Congo red was observed at low temperature (Fig. 2J and K), suggesting that IAs were involved in regulating the levels of ions and oxidants in the fungus at low temperature. It would be conceivable that IAs contributed to the fungal response to H_2_O_2_, because both 3–4 could be involved in reducing a H_2_O_2_ by donating a pair of hydrogen atoms. The presence of 1–2 in WT, which were dehydrogenated derivatives of 3–4, provided strong and direct evidence. Previous studies suggested that PIAs, analogues of 5–6, were involved in chelating iron to form insoluble Fe^3+^ complexes [18]. These iron chelators have been assumed to be involved in fungal iron uptake, which is considered as the major iron homeostatic mechanism in *A. fumigatus* and other *Aspergillus* species, and plays a crucial role in fungal pathophysiology [19]. We assumed that lack of PIAs as iron chelators might lead to increased free iron ions in Δ*NRPS* which might contribute to fungal response to infiltration of extracellular ion stress.

### 2.5 Decreased lipid droplets, enlarged vacuoles and shrunken mitochondria in Δ*NRPS* under cold stress

Transmission electronic microscopy (TEM) revealed that both Δ*NRPS* and WT at 37 °C had much more lipid droplets than their respective strains at 50 °C (Fig. 2L–M), indicating that low temperature could induce increased lipid levels in both strains. However, Δ*NRPS* displayed much less lipid droplets than WT at both temperatures (Fig. 2L), suggesting that lack of PIAs could lead to much less lipids in conidia. It seemed that the fungus at low temperature preferred to produce PIAs to accompany lipid accumulation, or lipid accumulation was inhibited by lack of PIAs, implying that only lipid accumulation without PIAs might bring detrimental impact for the fungus, consistent with previous finding that lipid accumulation was the paramount factor for ferroptosis induced by cold stress [8].

Careful observation of Δ*NRPS* conidia allowed us to distinguish a large bland area in the center of conidia (Fig. 2L). The infrastructures in mycelia at 37 °C became much clearer and brighter than in conidia (Fig. 2L–M). Vacuoles in Δ*NRPS* mycelia at 37 °C were much larger than those in WT mycelia at 37 °C (Fig. 2M). Moreover, 13.9 fold more myelin bodies were observed in the vacuoles of Δ*NRPS* at 37 °C while 12.3 fold more black tight bodies in those of WT at 37 °C (Fig. 2N). In fungi, Fe^2+^ that may bind with ferritin or phosphate and stored as complexes, such as ferrosomes, internal in vascular globoids as a protective mechanism against iron toxicity [20,21]. The black tight body, most likely ferrosomes, in those of WT. The lack of PIAs for sequestering iron might imposes additional iron storages in vacuoles, which became turgidly enlarged in Δ*NRPS*. This could explain why Δ*NRPS* displayed much stronger resistance to ion osmotic agent than WT likely ascribed to inner high iron levels in these enlarged vacuoles induced by lack of PIAs in Δ*NRPS.* Moreover, the mitochondria in most Δ*NRPS* mycelia at 37 °C were much small and in different shapes (white arrow, Fig. 2M), consistent with decreased lipid droplets for metabolism and iron homestasis disorder induced by lack of PIAs [22].

### 2.6 Decreased lipid level and ferroptosis in Δ*NRPS* under cold stress

**Fig. 3:**
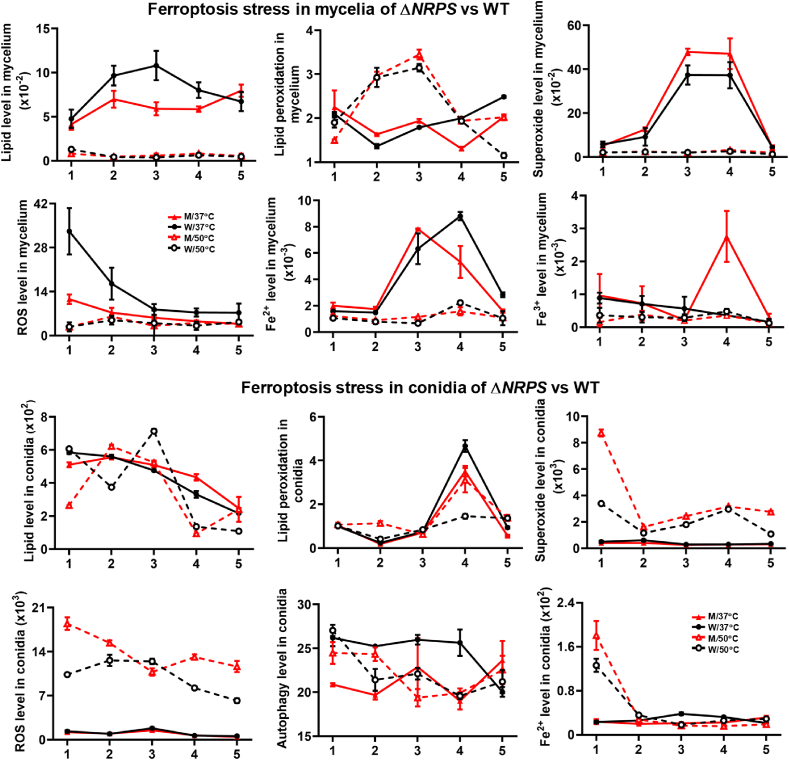
Effect of PIAs on lipid level and ferroptosis stresses in fungus under cold stress. Comparison of lipid level, lipid peroxidation level, superoxide level, ROS level and Fe^2+^ level, in both mycelia and conidia, together with Fe^3+^ level in mycelia and autophagy level in conidia between Δ*NRPS* (M) and WT at 37 °C and 50 °C.

Considering the different condition of Δ*NRPS* and WT, the conidia and mycelia were separated for close analysis. Due to the growth difference caused by different temperatures, all assays were performed on the same mycelial weight or the same spore number. Oil Red O Staining and neutral lipid assays also revealed that Δ*NRPS* and WT at 37 °C displayed dramatically higher lipid levels in mycelia than their corresponding strains at 50 °C, and Δ*NRPS* had significantly lower lipid level in mycelia than WT at 37 °C (Fig. 3), consistent with TEM result, confirming that low temperature could cause lipid accumulation and lack of PIAs might inhibit lipid level in Δ*NRPS*.

Interestingly, lipid peroxidation levels in both the mycelia and conidia of Δ*NRPS* were significantly lower than those in WT at 37 °C at day 4 (Fig. 3). Moreover, both Δ*NRPS* and WT at 37° displayed lower lipid peroxidation levels in mycelia than those at 50° during days 4–5, while the case was opposite in conidia at day 4. We also found that Δ*NRPS* at 37 °C displayed significantly increased superoxide level but decreased ROS level in mycelia compared with WT, while in conidia, both the superoxide and ROS levels in both Δ*NRPS* and WT at 37 °C were largely lower than those at 50 °C (Fig. 3). In particular, Δ*NRPS* conidia displayed decreased autophagy level than WT at 37 °C (Fig. 3). The decreased ferroptosis stress in Δ*NRPS* at 37 °C was consistent with the increased fungal growth on maize seeds and spore germination rate.

### 2.7 Function of PIAs in lowering Fe^3+^ level in fungus under cold stress

We then evaluated Fe^2+^ level in the conidia and mycelia of these two strains separately (Fig. 3). Both Δ*NRPS* and WT conidia at 50 °C displayed much higher Fe^2+^ level than those at 37 °C at day 1. Moreover, the Fe^2+^ level in Δ*NRPS* conidia was significantly higher than WT at 50 °C, suggesting that lack of PIA could lead to increased Fe^2+^ level in Δ*NRPS* conidia at 50 °C. However, the Fe^2+^ level in mycelia of Δ*NRPS* were quite similar in that of WT. Then we further evaluated Fe^3+^ and total iron level in mycelia (Fig. 3). At day 4, Δ*NRPS* at 37 °C displayed dramatically increased Fe^3+^ level compared with WT, consistent with large vacuoles in Δ*NRPS* for storing Fe^3+^. Further analysis exhibited that no significant difference in the total iron level between Δ*NRPS* and WT all the time (Fig. S4). The unchanged total iron level and increased Fe^3+^ level in Δ*NRPS* suggested that PIAs might be used to sequester Fe^3+^ as from lipid mass induced by a cold, instead of absorbing iron into fungi as previously described [18]. The PIAs secreting from *T. dupontii* might also sequester Fe^3+^ in the environment, which might block plant iron uptake for its growth, consistent with the result that Δ*NRPS* without PIAs outperformed WT in promoting maize sprout growth. Non-targeted metabolomics analysis of Δ*NRPS* and WT revealed that an ion peak at *m/z* 1038.53334 [M + H]^+^ for C_54_H_63_FeN_9_O_9_ (Fig. S5), most likely a trimeric complex of 5 derivative with Fe^3+^, similar to the previously described *Aspergillus terreus* metabolite, astechrome (C_60_H_66_FeN_9_O_9_, 1113.1, [M+H]^+^) [18] in the positive HRESI spectrum of WT, was disappeared in that of Δ*NRPS.* Previous study revealed that increased Fe^3+^ level might impair iron homeostasis which induced redox imbalance [8].

### 2.8 Accumulation of tryptophan degraded metabolites and free fatty acids in Δ*NRPS* under cold stress

Because gene *NRPS* was responsible for the condensation of tryptophan with glycine or alanine to form cyclic dipeptides from tryptophan with glycine or alanine, disruption of *NRPS* might lead to accumulation of these three amino acids in Δ*NRPS*. However, comparison of these amino acid levels in Δ*NRPS* and WT revealed that the levels of glycine and alanine were indeed increased in Δ*NRPS* vs WT at 37 °C while the level of trytophan significantly decreased (Fig. S6).

**Fig. 4:**
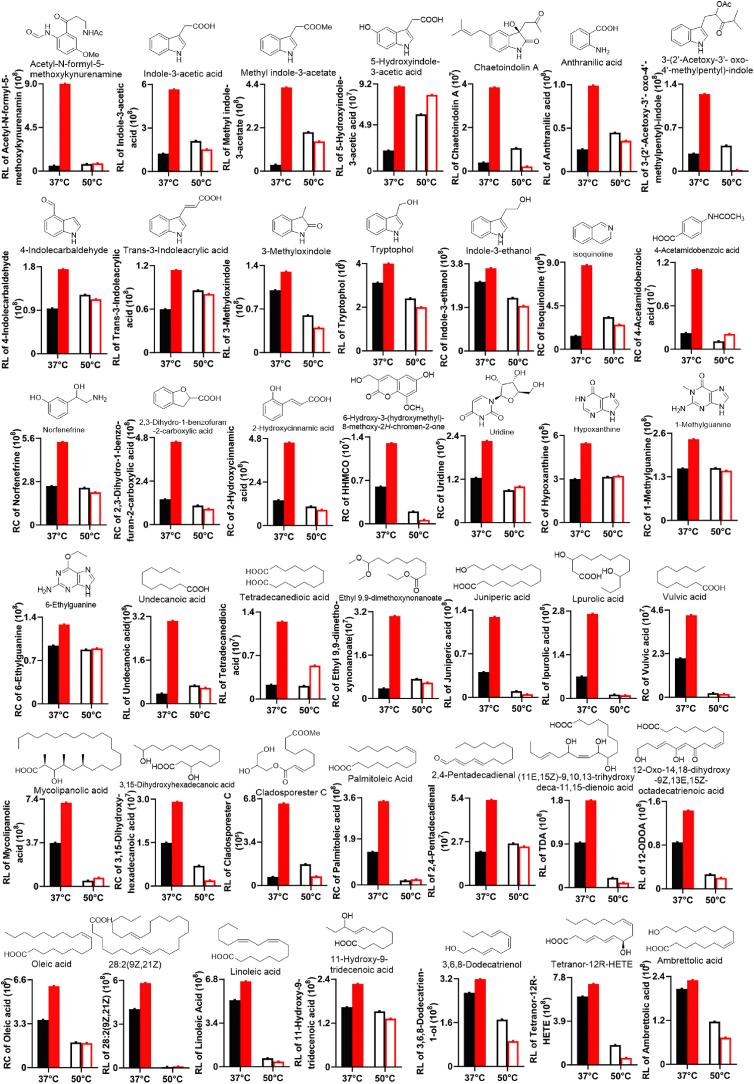
The most enriched 44 metabolites including 12 tryptophan-degraded compounds, 20 long chain free acids and 4 RNA-degraded base derivatives in Δ*NRPS* at 37 °C compared with Δ*NRPS* at 50 °C and WT at both temperatures.

Further non-targeted metabolics analysis of Δ*NRPS* and WT at 37 °C and 50 °C revealed that there were 42 compounds were most dramatically accumulated in Δ*NRPS* at 37 °C compared with Δ*NRPS* at 37 °C and WT at both temperatures (Tables S3 and S4). Interestingly, these compounds were mainly divided into two major types of metabolites, 22 aromatic metabolites and 20 free fatty acids (Fig. 4 and Fig. S7). Among 22 aromatic metabolites, 12 belonged to typical tryptophan-derived metabolites, including 10 indole derivatives, anthranilic acid, and kynurenamine derivative (Fig. 4 and Fig. S7). Among 20 free fatty acids, 11 were unsaturated and 9 were saturated. Saturated free fatty acids are the key precursors for triglycerides in lipid droplets (Fig. 4). Previous study suggested that saturated free fatty acids could be dehydrogenated or oxidized to unsaturated ones, such as via iron-mediated flavoprotein cytochrome *b*5 reductase [23]. Combined with the fact that increased Fe^3+^ level induced redox imbalance [24], we assumed that the saturated free fatty acids might be metabolized via an iron-mediated dehydrogenation/oxidation reaction to unsaturated ones, leading to the high levels of unsaturated free fatty acids in Δ*NRPS* at 37 °C. Previous study reported that free fatty acids, especially unsaturated fatty acids, such as ceriporic acid B, were induced by free radical reaction due to peroxidation [25,26], and the tryptophan derived metabolites including 3-hydroxyanthranilic acid and indoleacetic acid could also significantly lower plasma lipids, especially, indole-3-acetate could attenuate cytokine-mediated lipogenesis in hepatocytes [[27], [28], [29]]. Moreover, most recent study suggested that kynurenine could propagate anti-ferroptotic signaling because kynurenine was the source of molecules that suppressed redox cell death [30]. Ferroptosis is a non-apoptotic cell death featuring iron-dependent accumulation of lipid peroxidation [31]. All the highly enriched tryptophan derived metabolites and free fatty acids in Δ*NRPS* at 37 °C suggested that deficiency in PIAs caused increased Fe^3+^ level and then impaired redox homeostasis that induced the tryptophan and lipid metabolisms, which eventually led to suppressing ferroptosis by scavenging ROS and free radicals.

**Fig. 5:**
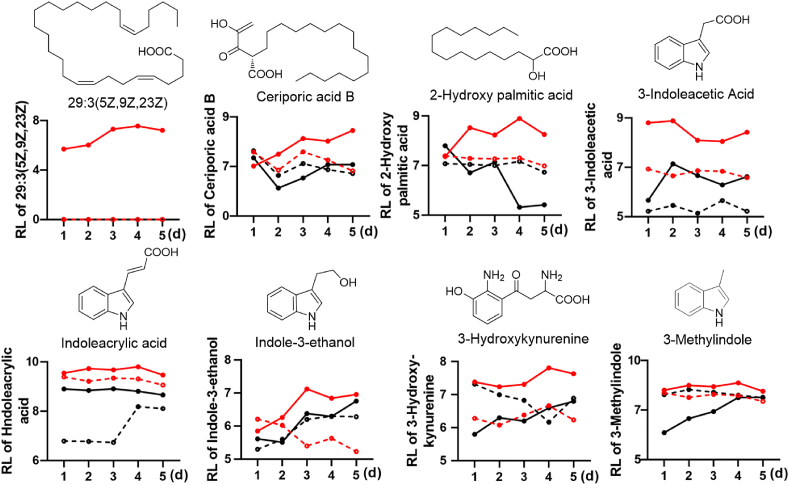
Comparison of the highly enriched key free acids and tryptophan-associated metabolites in Δ*NRPS* at 37 °C compared with Δ*NRPS* at 50 °C and WT at both temperatures for 5 days.

Further detailed analysis of these related key metabolites, including 29:3 (5Z,9Z,23Z), ceriporic acid B, 2-hydroxy palmitic acid, 3-indoleacetic acid, indoleacrylic acid, indole-3-ethanol, 3-hydroxykynurenine, and 3-methylindole in Δ*NRPS* and WT at both temperatures for 5 days revealed that all the compounds sharply increased in Δ*NRPS* vs. WT at 37 °C all the time (Fig. 5), suggesting that the highly enriched free fatty acids indeed coincided with highly increased tryptophan-derived metabolites in Δ*NRPS* at 37 °C.

### 2.9 Most up-regulated genes involved in tryptophan metabolism in Δ*NRPS* under cold stress

**Fig. 6:**
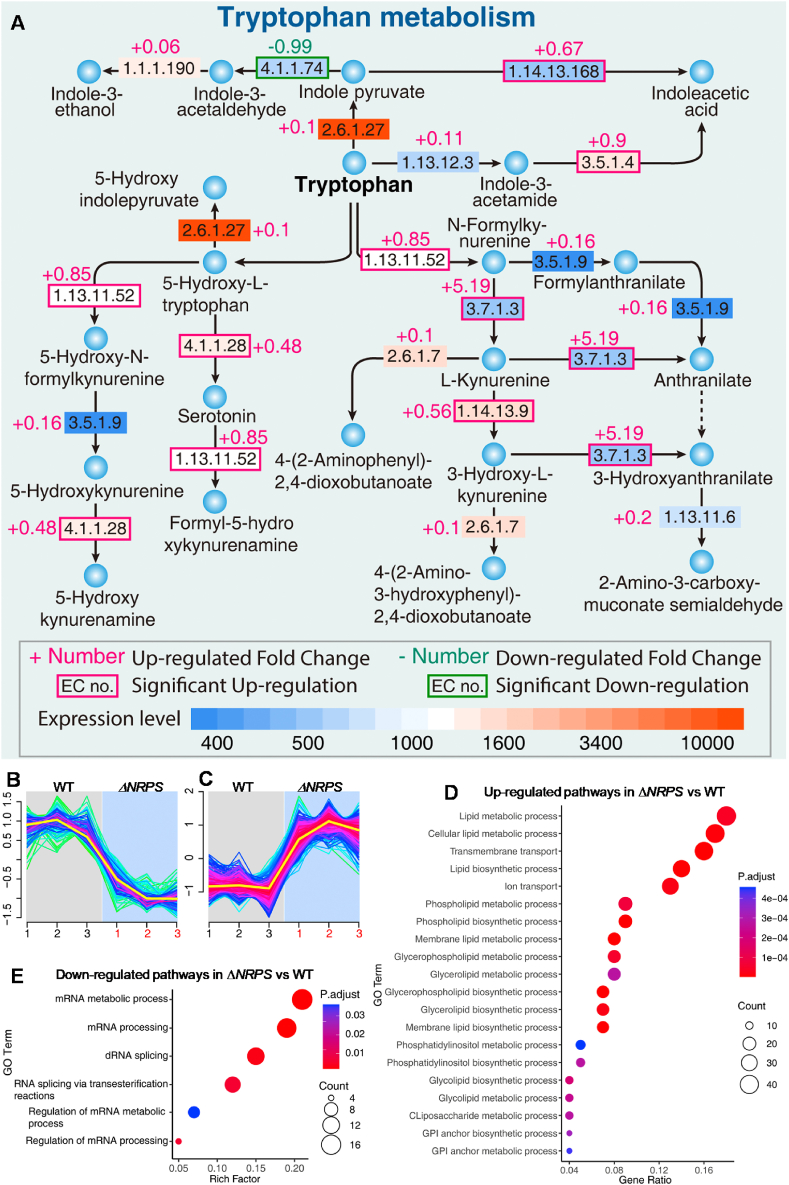
Transcriptional analysis illustrated that the most up-regulated key genes involved in tryptophan metabolism and pathways involved in lipid metabolic process. (A) All the genes crucial for tryptophan metabolism were significantly up-regulated in Δ*NRPS* vs. WT. (B–C) Clustering of the differentially regulated genes in Δ*NRPS* vs. WT under cold stress. (D–E) The up-regulated and down-regulated pathways in Δ*NRPS* vs. WT under cold stress.

Transcriptional analysis of Δ*NRPS* and WT at 37 °C revealed that 324 genes were significantly up-regulated and 124 genes were significantly down-regulated in Δ*NRPS* vs. WT (Tables S5 and S6, Fig. S8). The top 1 enriched gene *GME1605_g* in Δ*NRPS* vs WT at 37 °C was putatively assigned as kynureninase K01556, which catalyzes the cleavage of l-kynurenine (L-Kyn) and L-3-hydroxykynurenine (L-3OHKyn) into anthranilic acid (AA) and 3-hydroxyanthranilic acid (3-OHAA), respectively (Fig. 6A) [32]. Moreover, gene *GME1604_g*, just next to gene *GME1605_g*, which was putatively assigned as indoleamine 2,3-dioxygenase (IDO1), was also dramatically enriched in Δ*NRPS* vs WT. Enzyme indoleamine 2,3-dioxygenase is the first enzyme and rate limiting enzyme in the tryptophan catabolic pathway, which converts the l-tryptophan into N-formylkynurenine that is rapidly converted into kynurenine (Fig. 6A) [33]. In fact, all the genes crucial for tryptophan metabolism, were also significantly up-regulated in Δ*NRPS* vs. WT (Fig. 6A), consistent with highly accumulated tryptophan metabolites in Δ*NRPS* at low temperature. Previous studies suggested that the tryptophan-degrading reaction catalyzed by IDO1 was linked to ROS scavenging in Gr-1+/CD11b+ myeloid cell [34]. Kynurenine pathway, the major route of tryptophan catabolism, which is sensitive to redox environment and produces metabolites with redox capacity, which participate in redox reactions and their effect on cellular redox homeostasis [35]. Our result that *IDO1* was the most up-regulated gene in Δ*NRPS*, was consistent with dramatically enriched kynurenine derivative and the decreased ROS level in Δ*NRPS.*

The top 2 and 3 enriched genes *GME1861_g* and *GME166_g* in Δ*NRPS* vs. WT at 37 °C were putatively assigned as fungal trichothecene efflux pump (TRI12) and HCO_3_^−^ transporter family, respectively (Table S5) [36,37]. This was consistent with the increased tryptophan metabolism which might result in increased tryptophan-derived metabolites and one carbon unit [38].

### 2.10 Most up-regulated lipid metabolism and ion transport pathways in Δ*NRPS* under cold stress

The up-regulated KEGG pathways based on all the significantly up-regulated genes in Δ*NRPS* vs. WT at 37 °C (Fig. 6B–C), mainly involved lipid metabolic processes and ion transports, which predominantly top 15 up-regulated pathways in Δ*NRPS* vs. WT at 37 °C. Especially, the top 2 up-regulated pathways in Δ*NRPS* vs. WT were both lipid metabolic processes (Fig. 6D), consistent with highly enriched free fatty acids in Δ*NRPS*. The highly up-regulated ion transport in Δ*NRPS* vs. WT might be related the highly enriched iron ion in Δ*NRPS* due to lack of iron sequestering agent PIAs.

The most down-regulated pathways were mainly mRNA metabolic process and splicing, which were consistent with the most enriched guanine and its analogues and uridine in Δ*NRPS* vs. WT at 37 °C (Fig. 6E). Previous studies suggested that RNA was very vulnerable to oxidative damage due to its single stranded nature, and lack of an active repair mechanism for oxidized RNA, and especially these cytoplasmic RNAs were located in close proximity to the mitochondria where loads of ROS were produced [39]. This implied that increased oxidative stress due to deficiency in *NRPS* might cause damage to the mRNAs, then resulting in the decreased mRNA metabolic process and splicing.

## 3 Discussion

In this study, thermophilic fungus *T. dupontii* under cold stress that grew with stout conidiophores, accumulated not only lipid mass but also a class of tryptophan-derived PIAs. The deficiency in PIAs resulted in not only slim conidiophores, decreased lipids, shrunken mitochondria and enlarged vacuoles, but also largely enriched free fatty acids, tryptophan degraded metabolites and Fe^3+^ level. These results suggested that PIAs might have a multi-function as conidiophores binders in lowering the need for lipids, as lipid chaperones in sequestering Fe^3+^ from lipid mass, and as ROS scavenger in *T. dupontii* under cold-stress, consistent with previous study that lipid mass and Fe^3+^ were two key factors for “priming” of ferroptosis in thermophilic fungus *T. lanuginosus* [8]. Analysis of 2060 fungal genome sequences from 809 fungal genera revealed that 332 fungal strains corresponding to 155 fungal genera had analogues of the NRPS synthase genes required for PIA biosynthesis. Gene *NRPS* exists mainly in ascomyceta and basidiomyceta including 80 *Aspergillus* spp. (232 in total), 13 *Penicillium* spp. (44 in total), 2 *Trichoderma* spp. (27 in total), and 2 *Fusarium* spp (56 in total). This suggested that the role of PIAs in controlling lipid-mediated ferroptosis in cold stress adaption is common in fungal kingdom.

Interestingly, *T. dupontii* had an alternative strategy when PIAs failed to be formed under cold stress. Increased Fe^3+^ level due to lack of PIAs induced tryptophan and lipid metabolisms. A set of metabolites degraded from tryptophan were produced including potent ROS scavenger kynurenines, and a class of free radical scavengers, unsaturated free fatty acids were formed, which eventually led to suppressing ferroptosis by scavenging ROS and free radicals [31]. These results suggested that lipid mass and Fe^3+^ might be two key factors for “priming” of ferroptosis in the fungi under cold stress.

Tryptophan is a naturally-occurring essential amino acid, which has been advocated as an innocuous health food for the treatment of depression, insomnia, stress, behavioral disorders, and premenstrual syndrome, mainly due to its derived potent neuroactive substances, such as serotonin [33]. However, kynurenines are another type of tryptophan-degraded metabolites, which are known to result in immune suppression [33]. Interestingly, our results that kynurenine and 3-hydroxykynurenine were significantly increased in Δ*NRPS* vs. WT while serotonin was dramatically decreased under cold stress (Fig. S6), suggesting that without PIAs, cold stress could increase tryptophan-kynurenine pathway in the fungus. We then wondered if the fungal immune like system could be suppressed by the increased kynurenine pathway. Previous study suggested that in human, kynurenines should bind to the aryl hydrocarbon receptor (AHR) in multiple immune cell types, leading to immune suppression [39]. However, no AHR analogues were found in the fungus.

Previous study reported that a drop in temperature, such as felling by 5 °C, could suppress the human immune system to fight the cold virus, leading to catching more colds [40]. Because both cold stress and kynurenines have been reported to cause immune suppression in human, thus, if there is a link between temperature change and the tryptophan-kynurenine pathway in human, is worth investigating.

**Fig. 7:**
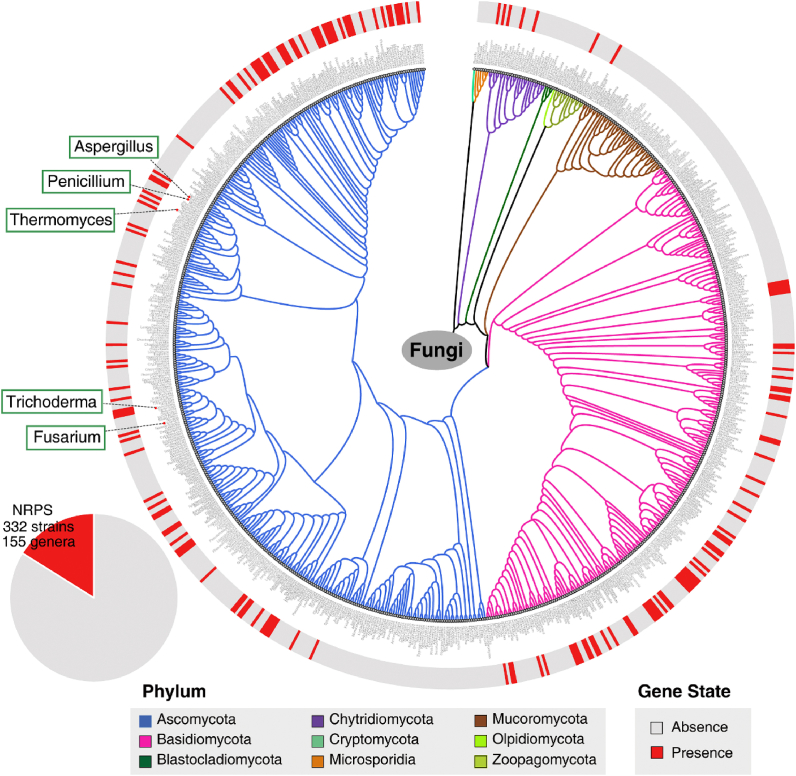
The phylogenetic tree of *NRPS* in 2062 fungal strains. Branches colors denote different fungal phylum information. The phylogenetic tree outside displays the state of a gene within a given genus. Red denotes presence of the gene in corresponding genus and gray denotes absence. (For interpretation of the references to color in this figure legend, the reader is referred to the Web version of this article.)

In summary, we found that a class of tryptophan-derived natural products, PIAs, accompanied the lipid formation in thermophilic fungus *T. dupontii* under cold stress, which might function as lipid chaperone in sequestering Fe^3+^ from lipid mass in *T. dupontii*. In the case that the biosynthetic step for PIAs was disrupted, tryptophan was metabolized to ROS scavengers and lipid inhibitors. Concurrently, a large amount of unsaturated and saturated free fatty acids, took the place of lipid mass in the mutant without PIAs. Our findings unveil two alternative metabolite-lipid-cold relationships (Fig. 7), and provide a mechanistic insight into the ecological and biological functions of tryptophan-associated metabolites in organisms.

### 3.1 Limitations of the study

Complementation of Δ*NRPS* with PIAs and ferroptosis-specific chemical inhibitors, Liproxstatin-1 (Lip-1) and Ferrostatin-1 (Fer-1), under cold stress will help better analysis in the fungus. Taking some mice cells under cold stress treated with PIAs at different temperatures and time points will help better analysis in other organisms.

## 4 Significance

We found that a common class of tryptophan-derived natural products, PIAs, were applied to accompany lipid formation in the fungus *T. dupontii* under cold stress, which might function as lipid chaperone in sequestering Fe^3+^ from lipid mass and lowering lipid in *T. dupontii*, because lipid mass and Fe^3+^ were two key factors for “priming” of ferroptosis. In the case that the biosynthetic step for PIAs was disrupted, redox sensitive tryptophan and lipid metabolisms were both induced by Fe^3+^-mediated redox homeostasis impairment. These results proved to be another evidence of a lipid-mediated ferroptosis in fungus under cold stress. This was the first reported case of the ecological function of tryptophan metabolism in organisms’ adaption to temperature changes. Our findings open a perspective for developing anti-cold strategy and agents.

## 5 Methods

### 5.1 Organisms, plasmids and culture conditions

*Thermomyces dupontii* was obtained from the State Key Laboratory for Conservation and Utilization of Bio-Resources & Key Laboratory for Microbial Resources of the Ministry of Education. *Saccharomyces cerevisiae* strain BJ5464-NpgA and plasmid pWX55 (a gift from Dr. Jingdan Liang, Shanghai Jiao Tong University) was used as host for assembly of DNA fragments. *Escherichia coli* strain DH5α (Tsingke, Beijing, China) was used to construct and store recombinational plasmids.

All fungal strains were cultivated on potato dextrose agar (PDA, potato (Kunming, China) 200 g/L, glucose (Solarbio, Beijing, China) 10 g/L, agar 15 g/L). About 9 mm inoculum of fungal strains were cultured on 9 cm diameter glass Petri dishes containing PDA plates at 50 °C for 7 days. Fungal media PDA was used for analysis of the colony growths and phenotypic traits of the fungal strains cultivated at 50 °C and 37 °C.

### 5.2 Metabolic analysis

Fungal strain *T. dupontii* was cultured on PDA for 7 days, and then 3–4 pieces of 5–6 mm mycelial disks were cut off and inoculated into each 500 mL flask containing 250 mL of potato dextrose broth (PDB, potato 200 g/L and glucose 10 g/L) and incubated at 50 °C and 37 °C, respectively, for 5 days on a rotary shaker (180 rpm). The fermentation broths were exhaustively extracted overnight with 250 mL ethyl acetate (1:1 v/v) and the organic layers were concentrated to dryness under reduced pressure. The dried organic residue was dissolved in 1 mL methanol, filtered through 0.22 μm membranes, and analyzed by HPLC-MS. HPLC-MS analysis were performed on a Q Extractive Focus UPLC-MS (Thermofisher, USA) with a PDA detector and an Obitrap mass detector (Shiseido, 5 μm, 4.6 × 250 mm, CAPCELL PAK C18 column) using positive and negative mode electrospray ionization. The total flow rate was 1 mL/min; mobile phase A was 0.1% formic acid in water; and mobile phase B was 0.1% formic acid in acetonitrile. The liquid chromatography (LC) conditions were manually optimized on the basis of separation patterns with the following gradient: 0–2 min, 10% B; 10 min, 25% B; 30 min, 50% B; 35 min, 90% B; 36 min, 95% B; 40 min, 95% B; 40.1 min, 10% B; and 45 min, 10% B. UV spectra were recorded at 196–400 nm. The data were analyzed and processed using Compound Discoverer 3.0 software (Thermofisher).

### 5.3 Transcriptional analysis

*T. dupontii* was cultured in PDB at 50 °C and 37 °C, respectively, for 5 days. Mycelia were collected by filtering the liquid culture and used for DNA extraction by using DNAiso Reagent (TaKaRa Biotechnology Co. Ltd, Dalian, China) as described in the manual.

RNA library was validating on the Agilent Technologies 2100 bioanalyzer for quality control. The double stranded PCR products above were heated denatured and circularized by the splint oligo sequence. The single strand circle DNA (ssCir DNA) was formatted as the final library. The final library was amplified with phi29 (Thermo Fisher Scientific, MA, USA) to make DNA nanoball (DNB) which had more than 300 copies of one molecular, DNBs were loaded into the patterned nanoarray and single end 50 bases reads were generated on Illumina platform (SeHealth Tech Co. Ltd, Wuhan, China). Three biological replicates were analyzed for each sample. Transcript abundances (FPKM) were provided by SeHealth Tech after sequencing and differentially expressed genes were calculated using R software. All genes with P-value ≤0.05 and log2 (fold_change) ≥ 1 were considered significantly differentially expressed.

### 5.4 Phylogenetic analysis

The genomes of fungal strain *T. dupontii* were analyzed using software antiSMASH (https://fungismash.secondarymetabolites.org/) to perform genome mining for the biosynthetic gene clusters. The NRPS genes were used as queries for BLASTP analysis in NCBI, and the protein sequence of the best hit (identity date > 50%) was selected for phylogenetic analyses. The neighbor-joining method was used for constructing evolutionary trees with MEGA X (2000 bootstrap replications).

### 5.5 T. dupontii mutant construction through homologous recombination

A modified protoplast transformation method for genetic disruption of target gene was applied using double-crossover recombination with the hygromycin-resistance gene (hygR) as a selection marker, followed by identification of desired mutants using diagnostic PCR. The two homologous regions were amplified from *T. dupontii* genomic DNA using primers containing overlapping regions with the vector pWX55 and the hygR cassette.

All primer sequences used are listed in Table S7. All the PCR products were amplified using GXL high-fidelity DNA Polymerase (TaKaRa Biotechnology Co. Ltd, Dalian, China). The 5′ flanks and 3′ flanks were amplified from the genomic DNA of *T. dupontii*, the hygromycin-resistance gene (hygR) was amplified from the pAg1-H3 vector by PCR. The DNA fragments (5′ flanks, 3′ flanks and hygR) were purified using PCR Clean-up Kit (Macherey-Nagel Inc, Düren, Germany) and NucleoSpin Gel, and were inserted into the specific sites of pWX55 vector. About 200 μg 5′ flanks and 3′ flanks and 100 μg pWX55 vector, which was digested by *XbaI* and *HindIII* restriction endonuclease, were transferred into 200 μL *Saccharomyces cerevisiae* BJ5464-*NpgA* chemically competent cells through polyethylene glycol mediated transformation method. And then these amplification products were assembled to form a 5′ flanks-hyg-3′ flanks cassette to generate the completed disruption vector by homologous recombination in yeast. The plasmid was extracted from yeast and then linearized with PCR. About 200 μL protoplasts (circa 1.0 × 10^8^/mL) were mixed with 10 μg linear DNA in a 1.5 mL centrifuge tube. After 40 min of incubation on ice, 1 mL of PTC (44.73 g/L KCl, 50 mM CaCl_2_, 50 mM Tris-HCl (pH 8.0), 60% polyethylene glycol 4000) was added into the mixture and mixed gently. After incubation at 45 °C for 30 min and 10 min of incubation on ice, the putatively transformed protoplasts were plated onto TB3 medium (200 μL/per plate) containing 200 μg/mL hygromycin B. Selections of transformation colonies and confirmation of the mutants deficient in target gene were performed following the above method for *T*. *dupontii* mutants.

### 5.6 Fungal morphology, conidial production, and germination

Mycelium plugs with an estimated 9-mm diameter from 7-day fungal colonies of the wild-type and mutant strains were inoculated onto 9-cm plates with several different media (5 replicates/strain) and then incubated at 45 °C. Radial colony growth diameter was measured every 2 days. Fungal strains were stained with calcofluor white (Sigma-Aldrich, St. Louis, MO, USA) and observed under a Zeiss Axioskop 2 Plus fluorescence microscope. To assess the sporulation capacities of the wild-type and mutant strains, 100-μL aliquots of suspensions of 10^6^ conidia were spread on 9-cm plates with PDA medium and incubated for 10 days at 45 °C. The conidia were washed in 10 mL of sterile water, followed by filtration through four layers of lens tissues to remove mycelium debris. The concentrations of the conidial suspensions were determined by microscopic counts on a hemocytometer and converted to the number of conidia per square centimeter of plate culture. The colonies of WTs and mutant strains were incubated on PDA medium at 50 °C for 6 days. The colonies were washed into 10 mL sterile water and filtered through six layers of lens tissue to remove hyphae. Then, 1 × 10^7^ spores were incubated in 1 mL liquid YPS medium at 50 °C for 6 h and at 37 °C for 12 h on a rotary shaker 180 rpm, respectively, to assay conidial germination rates.

### 5.7 Stress tolerance bioassays

The colonies of WT and mutant strains initiated with 9 mm diameter hyphal discs were incubated at 50 °C for 7 days on the plates of PDA alone (control) or supplemented with each of chemical stressors: (i) NaCl (0.3, 0.6, 0.9 mol/L) for osmotic stress; (ii) SDS (0.02%, 0.04%, 0.06%) and Congo red (0.1, 0.2, 0.3 mg/mL) for cell wall perturbation; and (iii) H_2_O_2_ (5, 10, 15 mmol/L) for oxidative stress [10]. Radial colony growth diameter was measured every 2 days.

### 5.8 Maize seed colonization assay

A maize kernel assay was performed according to a modified method as described study [41]. A mycelial plug for each strain was grown in 30 mL PDA liquid medium at 50 °C overnight, with shaking (180 rpm). Ten surface-sterilized maize (*Zea mays*) kernels were added to the overnight cultures, and samples were incubated at 37 °C for 144 h, 50 °C for 72 h. The maize kernels were incubated on filter paper, which was moistened daily to maintain humidity.

### 5.9 Scanning electron microscopy

To examine conidial morphology, conidia were harvested from 7 day at 50 °C and 14 day at 37 °C, respectively, fungal WT and mutant cultures grown on PDA. The number of conidia and their morphologies were next determined and observed as previously described [42]. After critical-point drying using a model CPD-030 device (Bal-Tec AG, Balzers, Liechtenstein), the samples were coated with gold using a model SCD 005 sputter coater (Bal-Tec) and observed by scanning electron microscopy (Quanta-200; FEI, Hillsboro, OR, United States).

### 5.10 Transmission electron microscopy

The ultrastructural features of fungal WT and mutant cultures were examined using transmission electron microscopy (TEM). Fresh mycelia were cultured in PDB for 24 h at 50 and 37 °C, respectively to remove any medium, and then were incubated with 2.5% glutaraldehyde (Sigma) in phosphate buffer (pH 7.4) overnight at 4 °C. Conidia were cultured in PDA for 7day at 50 °C and 14 day at 37 °C. The samples were pretreated in 4% paraformaldehyde, then processed with 2.5% glutaraldehyde (Sigma) in phosphate buffer (pH 7.4) overnight at 4 °C and post-fixation in 1% OsO4, the samples were dehydrated in a graded ethanol series, embedded in Spurr resin, and stained with 2% uranyl acetate and Reynold's lead solution. The samples were examined under an H-7650 transmission electron microscope (Hitachi).

### 5.11 Neutral lipid assay

Lipid Assay Kit (#ab242307, Abcam) was used for neutral lipid detection assay, and this kit was firstly used for evaluation of neutral lipid level in fungi. In brief, harvested spores (1 × 10^7^) from PDA were treated with the 1 × fluorometric reagent (diluted with 200 μL cold water) for 30 min at room temperature protected from light in 1.5 mL centrifuge tube. After the stained spores were washed three times in cold water, the fluorescence intensities were measured at Ex/Em = 490 nm/585 nm by flow cytometry (BD Biosciences, USA), and the data were analyzed using FlowJo VX software (BD Biosciences, USA) [8].

### 5.12 ROS/superoxide assay

ROS/Superoxide Detection Assay Kit (#ab139476, Abcam) was used to directly monitor real time production of reactive oxygen species (ROS) in mycelia or spores using flow cytometry (spores) or microplate reader (mycelia)with minor modifications. This kit was firstly used for evaluation of ROS level in Fungi. Briefly, mycelia (20–80 mg) or spores (1 × 10^7^) were harvested from PDA in 1.5 mL centrifuge tube and were treated with 500 μL of ROS/Superoxide Detection Solution, and then incubated for 60 min at 37 °C or 50 °C in the dark. The stained spores were analyzed by flow cytometry and the data were analyzed using FlowJo VX software. The stained mycelia were analyzed by microplate reader. Oxidative Stress Detection Reagent (Green, Ex/Em 490/525 nm) was used for evaluation of total ROS, and Superoxide Detection Reagent (Orange, Ex/Em 550/620 nm) for Superoxide [43].

### 5.13 Lipid peroxidation detection assay

Image-iT® Lipid Peroxidation Kit (#C10445, Life Technologies) was used for evaluation of lipid peroxidation level in fungi [8]. In our experiment, spores (1 × 10^7^) were harvested from PDA in 1.5 mL centrifuge tube and were treated with 200 μL phosphate buffered saline (PBS) containing 5 μM C11-BODIPY 581/591. Spores were incubated for 30 min at 37 °C and then suspended in 500 μL PBS and subjected to flow cytometry analysis. The lipid peroxidation was determined by evaluating the fluorescence intensities and calculating the ratio of intensity in green channel (∼510 nm) to the intensity in red channel (∼591 nm) per spore. For analysis of lipid peroxidation in mycelia, Lipid Peroxidation (MDA) Assay Kit (Colorimetric/Fluorometric) (#ab118970, Abcam) was used according to the manufacturer's instructions with minor modifications, and this kit was firstly used for evaluation of lipid peroxidation level in Fungi [44]. Briefly, mycelia (20–80 mg) were harvested from PDA in 1.5 mL centrifuge tube. After washed in cold PBS, mycelia were homogenized in 303 μL lysis solution (buffer + BHT) with a dounce homogenizer sitting on ice with 50–100 passes. Then added 600 μL of TBA solution to the mycelia for incubating at 95 °C for 60 min and cooled in ice bath for 10 min. Finally, transferred 200 μL supernatant to wells of microplate and measured the fluorescence intensities at OD = 532 nm on microplate reader.

### 5.14 Iron measurement

Iron assay kit (#ab83366, Abcam) was used for evaluation of intracellular ferrous iron level (Fe^2+^ and Fe^3+^) in fungi [44]. Firstly, mycelia (20–80 mg) were collected in PBS, and homogenized in iron assay buffer using a Dounce homogenizer sitting on ice with 50–100 passes, then iron reducer was added in to the collected supernatant, mixed, and incubated. Finally, iron probe was added, mixed, and incubated for 1 h, and the incubated solution was immediately measured on microplate reader at OD = 593 nm.

In the process of ferroptosis, the iron-regulatory pathway is hijacked through multiple mechanisms to increase intracellular labile iron, and the intracellular labile iron is a major contributor to the lipid peroxidation because of its ability to produce harmful reactive oxygen species via contact with oxygen, superoxide, and hydrogen peroxide (H_2_O_2_) [8]. Besides, the spores of *T. lanuginosus* are too small and hard to homogenize properly. Therefore, we evaluated intracellular labile iron level in spores using BioTracker Far-red Labile Fe^2+^ Dye (#SCT037, Merck) through flow cytometry analysis according to the manufacturer's instructions (Alonzi et al., 2019), and this kit was firstly used for evaluation of labile Fe^2+^ content in Fungi. Briefly, harvested spores (1 × 10^7^) from PDA were treated with 5 μM BioTracker Far-red Labile Fe^2+^ Dye (diluted with 200 μL cold water) for 90 min at room temperature protected from light in 1.5 mL centrifuge tube. After the stained spores were washed three times in cold water, the fluorescence intensities were measured at Ex/Em = 646 nm/662 nm with flow cytometry.

### 5.15 Evolutionary analysis

To inspect the universal existence of these key genes in fungi, we search the protein sequence of each gene against gene catalog protein in MycoCosm database using BLASTP algorithm. Only the alignment hit coverage (%) and sequence identity (%) of a sequence greater than 40 was retained, and the fungi species possessing this sequence was deemed to have a corresponding gene. Setting up a legible background can help us to display the distribution of these genes. A phylogenetic tree of 749 fungal genera was built according to the topological structure of the fungal tree of life in PhycoCosm using ‘nwk’ format. All the information was mapped to the fungal phylogenetic tree (genus level) in MycoCosm to show the presence or absence of each gene. Phylogenetic tree visualization was manipulated in iTol software (https://itol.embl.de/).

### 5.16 Statistical analyses

Data from three biological repeated experiments were expressed as means ± SD, which were analyzed by one-way analysis of variance followed by Tukey's multiple comparison test, with *p* values < 0.05, *, *p* values < 0.01, **, *p* values < 0.001, ***, considered statistically significant. All statistical analyses were conducted using GraphPad Prism ver. 8.2.1 for Windows (GraphPad Software, San Diego, CA, USA).

## Author contribution statement

Keqin Zhang: contributed to reagents, materials, analysis tools, or data.

Shenghong Li: analyzed the data; contributed to reagents, materials, analysis tools, or data; wrote the manuscript.

Xuemei Niu: conceived the study and designed experiments; analyzed the data; contributed to reagents, materials, analysis tools, or data; wrote the manuscript.

Yonghong Chen: performed the experiments; analyzed the data; contributed to reagents, materials, analysis tools, or data; wrote the manuscript.

Xiaoyu Yang: performed the experiments; analyzed the data; contributed to reagents, materials, analysis tools, or data; wrote the manuscript.

Longlong Zhang: performed the experiments; analyzed the data; contributed to reagents, materials, analysis tools, or data; wrote the manuscript.

Qunfu Wu: performed the experiments; analyzed the data; contributed to reagents, materials, analysis tools, or data; wrote the manuscript.

Shuhong Li: performed the experiments; contributed to reagents, materials, analysis tools, or data.

JiangHui Gou: performed the experiments; contributed to reagents, materials, analysis tools, or data.

Jiangbo He: performed the experiments; contributed to reagents, materials, analysis tools, or data.

## Funding statement

This Xuemei Niu was supported by 10.13039/501100001809National Natural Science Foundation of China [21977086]; National Natural Science Foundation of China-Yunnan Joint Fund [21867018].

## Data availability statement

All data and methods necessary to reproduce this study are included in the manuscript and Supplemental Information. All transcriptomic data will be available via NCBI (https://www.ncbi.nlm.nih.gov) with accession number PRJNA841250.

## Declaration of interest's statement

The authors declare that they have no known competing financial interests or personal relationships that could have appeared to influence the work reported in this paper.
