# Supplementary material for: Tryptophan-centered metabolic alterations coincides with lipid-mediated fungal response to cold stress
Source: Heliyon. 2023 Jan 21;9(2):e13066. doi: 10.1016/j.heliyon.2023.e13066 (PMC9898655; doi:10.1016/j.heliyon.2023.e13066)
Supplement: Multimedia component 1 [file mmc1.docx]

Supplementary Information

**Tryptophan-Centered Metabolic Alterations Coincides with Lipid-Mediated Fungal Response to Cold Stress**

Yonghong Chen^1,4^, Xiaoyu Yang^1,4^, Longlong Zhang^1,4^,Qunfu Wu^1,4^, Shuhong Li^1^, Jiang-Hui Gou^1^, Jiangbo He^3^, Keqin Zhang^1^, Shenghong Li^2*^ and Xuemei Niu^1,5*^

^1^State Key Laboratory for Conservation and Utilization of Bio-Resources in Yunnan, and Key Laboratory for Southwest Microbial Diversity of the Ministry of Education, Yunnan University, Kunming 650032, China

^2^ State Key Laboratory of Phytochemistry and Plant Resources in West China, Kunming Institute of Botany, Chinese Academy of Sciences, Kunming 650204, P. R. China

^3^Kunming Key Laboratory of Respiratory Disease, Kunming University, Kunming 650214, P. R. China Kunming Key Laboratory of Respiratory Disease

^4^These authors contributed equally

^5^Lead contact

*Correspondence: [xmniu@ynu.edu.cn](mailto:xmniu@ynu.edu.cn) (Xuemei Niu), [shli@cdutcm.edu.cn](mailto:shli@cdutcm.edu.cn) (Shenghong Li)


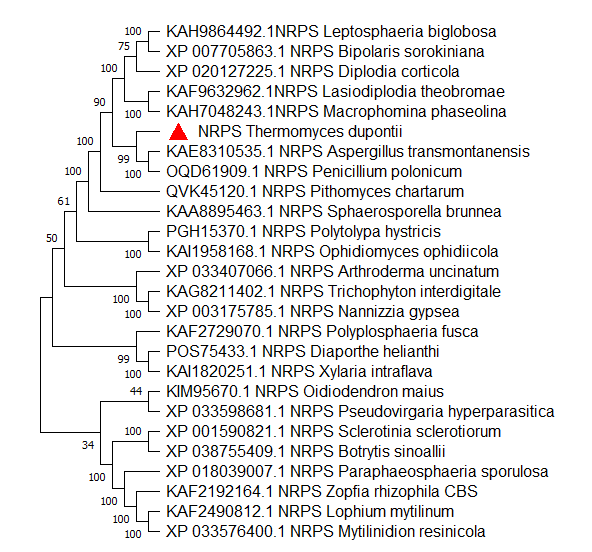


Figure S1. The phylogenetic tree of NRPS

Figure S2. Schematic of homologous recombination of *NRPS*; Confirmation of the mutant Δ*NRPS* by PCR analysis (line 7: genomic DNA from mutant Δ*NRPS*, M: DL 5000 DNA Marker, WT: plasmid DNA as control ).

Figure S3. HPLC-MS profiles of the fermentations of WT (black line) and mutant Δ*NRPS*.


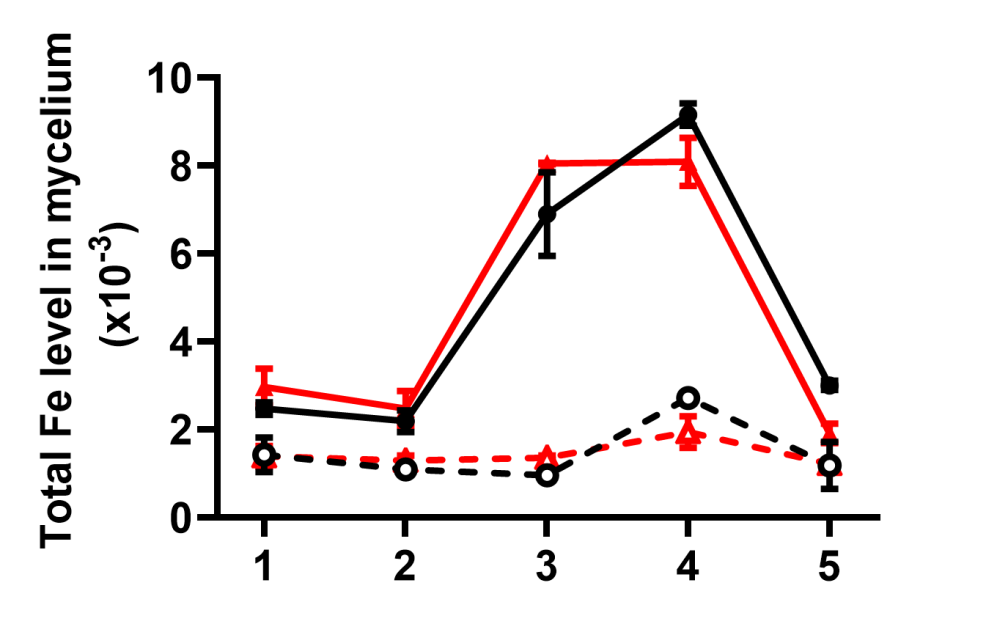


**M/37°C**

**W/37°C**

**M*/*50°C**

**W/50°C**

Figure S4. Comparison of total iron level in mycelia between the PIA-negative mutant Δ*NRPS* (M) and WT at 37°C and 50°C.

Figure S5. Comparison of an ion peak at *m/z* 1038.53334 [M+H]^+^ in the positive HRESI spectra between the PIA-negative mutant Δ*NRPS* (M) and WT at 37°C.

Figure S6. Comparison of the levels of PIA precursors between the PIA-negative mutant Δ*NRPS* (red) and WT (black) at 37°C and 50°C, including tryptophan, lipid alanine, and glycine.

Figure S7. The aromatic compounds in the most enriched metabolites in Δ*NRPS* (red) vs WT (black) at 37°C and 50°C.


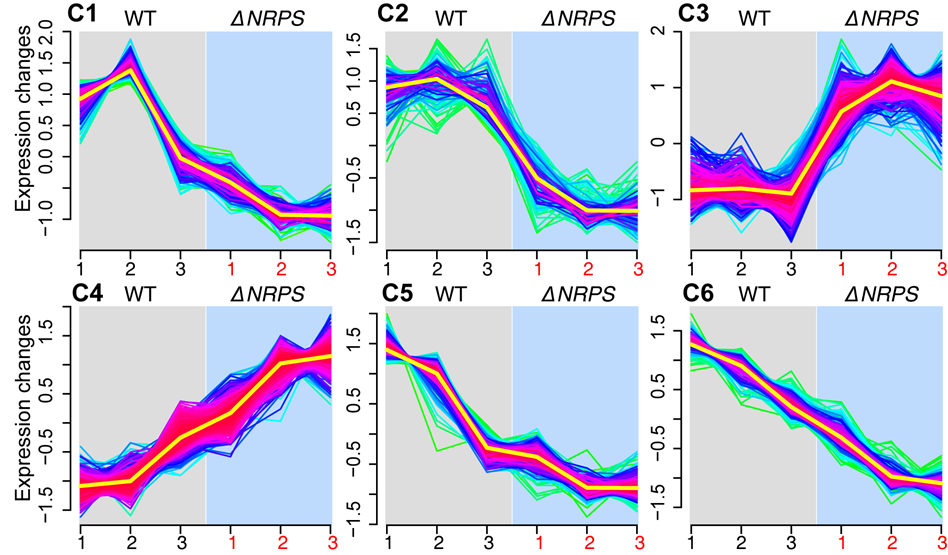


Fig S8. Clusters of genes with similar expression patterns among samples using the fuzzy c-means algorithm. X axis of line plots show normalized (log) gene expression change, X axis represent samples in WT and Δ*NRPS*, the orange line representing the values for the cluster center. Fuzzy c-means clustering of genes expression among was performed using the Mfuzz package in the statistical programming language R.

Table S1. The list of top 20 up-regulated genes of *T. dupontii* grown at 37℃ compared with those at 50℃ (P<0.05)

| No. | Gene ID | log2FC (37°C/50°C) | Scaffold | Function |
| --- | --- | --- | --- | --- |
| 1 | GME378_g | 7.230993 | scaffold_1 | Hypothetical protein |
| 2 | GME3114_g | 7.165595 | scaffold_4 | Hypothetical protein |
| 3 | GME4766_g | 6.970967 | scaffold_8 | Aromatic prenyltransferase |
| 4 | GME375_g | 6.951284 | scaffold_1 | Conidial pigment biosynthesis oxidase Arb2 |
| 5 | GME3593_g | 6.702929 | scaffold_5 | Xylose isomerase-like superfamily |
| 6 | GME376_g | 6.603795 | scaffold_1 | Hypothetical protein |
| 7 | GME290_g | 6.539638 | scaffold_1 | Conidial hydrophobin Hyp1/RodA |
| 8 | GME4768_g | 6.501188 | scaffold_8 | MFS transporter |
| 9 | GME379_g | 6.073363 | scaffold_1 | Conidial pigment biosynthesis oxidase Abr1/brown 1 |
| 10 | GME281_g | 6.054659 | scaffold_1 | Hypothetical protein |
| 11 | GME4771_g | 6.028753 | scaffold_8 | Aromatic prenyl-transferase |
| 12 | GME6122_g | 6.02837 | scaffold_12 | Transcription factor AbaA |
| 13 | GME377_g | 5.94731 | scaffold_1 | Scytalone dehydratase |
| 14 | GME2987_g | 5.856978 | scaffold_4 | NA |
| 15 | GME4009_g | 5.792565 | scaffold_6 | lycosyl hydrolases |
| 16 | GME4770_g | 5.576828 | scaffold_8 | AMP-binding enzyme |
| 17 | GME4079_g | 5.362232 | scaffold_6 | Putative threonine/serine exporter |
| 18 | GME6256_g | 5.35643 | scaffold_13 | NA |
| 19 | GME7089_g | 5.323864 | scaffold_60 | NA |
| 20 | GME4769_g | 5.321371 | scaffold_8 | P450 monooxygenase |

Table S2. The list of top 10 down-regulated genes of *T. dupontii* grown at 37℃ compared with those at 50℃ (P<0.05)

| No. | Gene ID | log2FC (37°C/50°C) | Scaffold | Function |
| --- | --- | --- | --- | --- |
| 1 | GME2140_g | -7.60057 | scaffold_3 | Bestrophin |
| 2 | GME1858_g | -5.84453 | scaffold_3 | 2,6-dihydropseudooxynicotine hydrolase |
| 3 | GME1806_g | -5.78312 | scaffold_2 | FAD-binding |
| 4 | GME3684_g | -4.47442 | scaffold_5 | NA |
| 5 | GME2856_g | -4.45273 | scaffold_4; | Methyltransferase |
| 6 | GME1856_g | -4.33871 | scaffold_3 | NA |
| 7 | GME3070_g | -4.22861 | scaffold_4 | Hypothetical protein |
| 8 | GME5700_g | -4.08209 | scaffold_10 | NA |
| 9 | GME1326_g | -3.96668 | scaffold_2 | S-adenosyl-L-methionine-dependent methyltransferase |
| 10 | GME1353_g | -3.96668 | scaffold_2 | S-adenosyl-L-methionine-dependent methyltransferase |
| 11 | GME4731_g | -3.95733 | scaffold_8 | Alpha-N-acetylglucosaminidase |
| 12 | GME2972_g | -3.90309 | scaffold_4; | Alpha-amylase |
| 13 | GME1861_g | -3.86187 | scaffold_3 | MFS transporter superfamily |
| 14 | GME1864_g | -3.765 | scaffold_3 | Aldehyde dehydrogenase |
| 15 | GME4634_g | -3.75274 | scaffold_7 | Phosphotransferase |
| 16 | GME1857_g | -3.70863 | scaffold_3 | Nonribosomal peptide synthetase |
| 17 | GME6524_g | -3.70663 | scaffold_14 | S-adenosyl-L-methionine-dependent methyltransferase |
| 18 | GME6410_g | -3.68756 | scaffold_13 | Hypothetical protein |
| 19 | GME2702_g | -3.61167 | scaffold_4 | Sugar transporter |
| 20 | GME276_g | -3.60578 | scaffold_1 | Phosphoinositide phospholipase C, Ca2+-dependent |

Table S3. The up-regulated metabolites in Δ*NRPS* and WT at 50°C and 37°C. (See excel material, P<0.05).

Table S4. The down-regulated metabolites in Δ*NRPS* and WT at 37°C. (See excel material, P<0.05).

Table S5. The up-regulated genes in Δ*NRPS* and WT at 37°C. (See excel material, P<0.05).

Table S6. The down-regulated genes in Δ*NRPS* and WT at 37°C. (See excel material P<0.05).

Table S7. The primers were used in this study.

| Primer name | Sequence (5' to 3') |
| --- | --- |
| Hyg F  Hyg R | TCTGTCTCTTGGCTGCCCTCCTCGACATCGATGATCAGGCCTCGACAG  GCGGATCGGAAGACGTTCGCTTGCTTCGGGGGATCCTCTAGATCTCGAC |
| NRPS-up-F  NRPS-up-R  NRPS-down-F  NRPS-down-R | TGATGATGATAAGACTAGTCGATCAGTGCGTGGTGAGC  TCAATATCATCTTCTGTCGAGGTGGGCTCTCGGCGAAG  TTAGAGGTAATCCTTCTTTGTCTTTTCTCCGTGCCGACG  TGATGGTGATGCACGTGCAAGTGAGCAAGATTAACGGAG |
| NRPS-verify-F  NRPS-verify -R | CGTAATCTTCGCCGAGAGC  TGTACCACCTCGTCGGCAC |
